# An early-stage adenocarcinoma originating from a 15-cm long-segment Barrett’s esophagus: A case report

**DOI:** 10.1097/MD.0000000000047251

**Published:** 2026-01-23

**Authors:** HaoQiang Zhi, Miao Wang, Ling Sun, WenJing Ao, WeiJian Ju, Quan Man, HuiFang Pang

**Affiliations:** a Department of Gastroenterology, Affiliated Tongliao Clinical Medical College of Inner Mongolia Medical University, Tongliao, Inner Mongolia, China; b Department of Digestive Endoscopy Center, Tongliao People’s Hospital, Tongliao, Inner Mongolia, China; c Department of Gastroenterology, Tongliao People’s Hospital, Tongliao, Inner Mongolia, China; d Department of General Surgery, Tongliao People’s Hospital, Tongliao, Inner Mongolia, China.

**Keywords:** Barrett’s esophagus, case report, confocal laser endomicroscopy, esophageal adenocarcinoma, sponge cytology

## Abstract

**Rationale:**

Barrett’s esophagus (BE), a precancerous lesion for esophageal adenocarcinoma, is classified into short-segment and long-segment types, with the latter being less common. This report describes a rare case of early-stage adenocarcinoma originating from a long-segment BE, approximately 15 cm from the gastro-esophageal junction.

**Patient concerns:**

A 68-year-old male presented with intermittent acid reflux and heartburn for over 30 years, which had worsened over the past month, accompanied by discomfort during eating. Gastroscopy identified a flat lesion measuring approximately 1.5 cm × 1.0 cm, located 30 cm from the incisors. Pathological examination suggested high-grade adenomatous dysplasia with possible carcinoma.

**Diagnoses:**

Long-segment BE; flat-type esophageal lesion (0-IIb + 0-IIc) with high-grade adenomatous dysplasia and focal well-differentiated adenocarcinoma, suspected to invade the muscularis mucosae layer.

**Interventions:**

Following preoperative evaluation, the endoscopic submucosal dissection procedure was successfully performed under general anesthesia, The preoperative diagnosis and postoperative surveillance of this patient also incorporated the use of a cytosponge™ device and confocal laser endomicroscopy.

**Outcomes:**

The patient remained free of tumor recurrence or metastasis during subsequent follow-up visits.

**Lessons:**

Endoscopic submucosal dissection is an effective treatment for early-stage esophageal adenocarcinoma, early detection and treatment are key to improving survival, cytosponge™ device helps to detect high-risk groups. Confocal laser endomicroscopy represents an advanced endoscopic imaging modality that facilitates preoperative diagnosis and postoperative surveillance in early-stage esophageal carcinoma.

## 1. Introduction

Esophageal cancer ranks as the 11th most common cancer and the seventh leading cause of cancer-related deaths worldwide, posing a significant global health challenge.^[1]^ It has 2 major subtypes: esophageal squamous cell carcinoma (ESCC) and esophageal adenocarcinoma (EAC). Regardless of subtype, prognosis remains poor, as most cases are diagnosed at advanced stages, underscoring the critical need for early detection. Barrett’s esophagus (BE) is characterized by the replacement of esophageal squamous epithelium with columnar epithelium, extending at least 1 cm from the gastro-esophageal junction. Its development is associated with gastro-esophageal reflux disease (GERD), with approximately 15% of patients with GERD progressing to BE.^[2]^ BE is the only established precancerous lesion for EAC. It is classified into short-segment and long-segment types, with the latter being less common but carrying a higher risk of progression to dysplasia or adenocarcinoma.^[3]^ Clinically, long-segment BE with adenocarcinoma is rare, and reports of cases involving a metaplasia length of 15 are even scarcer. Traditionally, early-stage esophageal cancer has been treated with esophagectomy. However, endoscopic treatments have gained preference due to their minimal invasiveness, safety, and effectiveness. Unlike surgery, endoscopic procedures preserve the physiological structure of the esophagus, further emphasizing the importance of early diagnosis. However, early-stage esophageal cancer lacks distinct clinical symptoms and reliable diagnostic biomarkers, making upper gastrointestinal endoscopy the primary screening tool. Despite its effectiveness, endoscopy is limited by procedural complexity and resource constraints, making large-scale screening in high-risk populations impractical. Thus, there is an urgent need for a safe, accessible, and effective alternative.

The cytosponge™ device, a recently developed esophageal cell collection device, offers a promising solution. When combined with artificial intelligence-assisted cytological analysis that utilizes machine learning models to detect abnormal cells, this device has demonstrated satisfactory diagnostic accuracy. Probe-based confocal laser endomicroscopy (CLE) is an endoscopic technique that enables in vivo histological evaluation using fluorescent pigment.

In this case, a combination of cytosponge™ device screening, endoscopy, and histopathology confirmed the diagnosis and the patient underwent successful endoscopic submucosal dissection (ESD), achieving favorable postoperative recovery. By reporting this case, we aim to provide valuable insights into the diagnosis and treatment of early-stage esophageal cancer. We present this case in accordance with the CARE reporting checklist.

## 2. Case presentation

### 2.1. Chief complaint

The patient is a 68-year-old male, he presented with intermittent acid reflux and heartburn for over 30 years, which had worsened over the past month, accompanied by discomfort during eating.

### 2.2. History of present illness

The patient first experienced acid reflux and heartburn symptoms over 30 years ago, with intermittent episodes that were not considered severe. Three years ago, a gastroscopy revealed BE, for which the patient received acid-suppressive therapy, leading to symptom relief. However, 1 month before admission, the symptoms worsened, with increasing discomfort while eating. A repeat gastroscopy identified a flat lesion measuring approximately 1.5 × 1.0 cm, located 30 cm from the incisors. Pathological examination suggested high-grade adenomatous dysplasia with possible carcinoma. Seeking further evaluation and treatment, the patient was admitted to our hospital with a diagnosis of “atypical epithelial hyperplasia of the esophagus.”

### 2.3. History of past illness

The patient had a history of hypertension for 5 years and diabetes for 6 years.

### 2.4. Personal and family history

The patient had no history of smoking or alcohol consumption and no known family history of gastrointestinal malignancies.

### 2.5. Physical examination

Upon admission, the patient’s vital signs were as follows: body temperature, 36.5℃; pulse, 66 beats/min; respiratory rate, 20 breaths/min; and blood pressure, 144/91 mm Hg. No other abnormalities were noted on physical examination.

### 2.6. Laboratory examinations

Liver function: normal, blood glucose: 6.7 mmol/L, glycosylated hemoglobin: 11.6%, total protein 63.2 g/L.

### 2.7. Cytological examination

**Figure 1.**
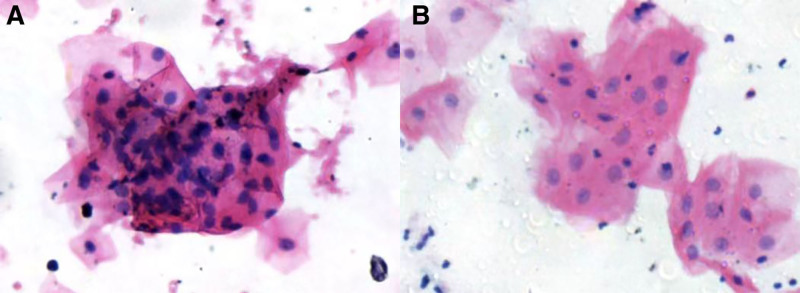
Cytological findings (Feulgen-eosin stain, ×200). (A) Preoperative cytology showing atypical cells; (B) postoperative cytology indicating squamous cell hyperplasia.

Cytological examination performed 1 day prior to surgery revealed atypical cells on cytopathological analysis (Fig. 1A).

### 2.8. Imaging examinations

**Figure 2.**
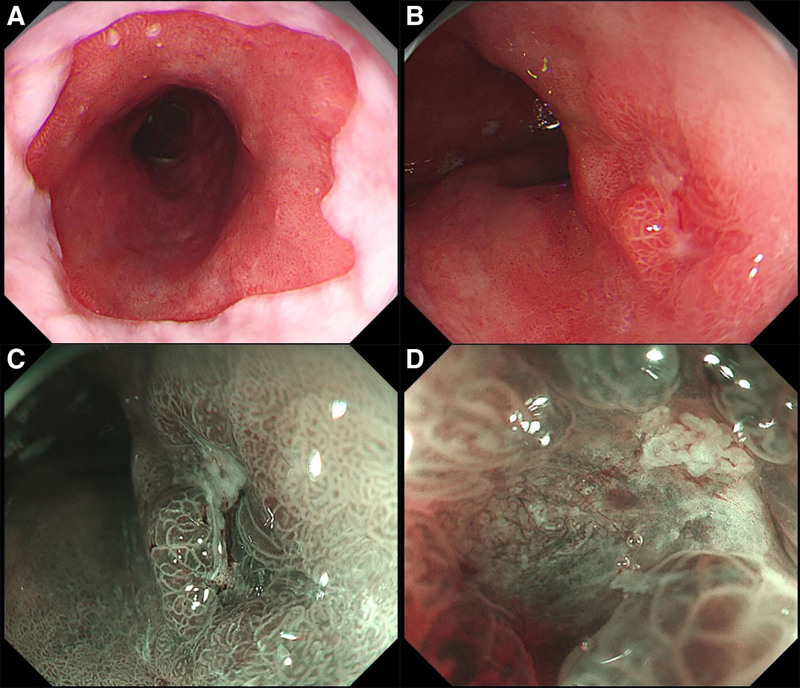
Magnified endoscopy findings. (A) Barrett’s esophagus extending 15 cm in length; (B) early esophageal cancer lesion observed under white light endoscopy; (C) early esophageal cancer lesion visualized using narrow-band imaging; (D) early esophageal cancer lesion examined under magnifying endoscopy.

Magnified endoscopy: A flat lesion measuring approximately 1.8 cm × 1.2 cm was observed on the right posterior wall, extending from approximately 20 to 35 cm from the incisors. The lesion exhibited a rough, reddish surface with localized indentation and erosion, partially covered with a thin white film (Fig. 2A, B).

Narrow-band imaging with magnification endoscopy (NBI-ME): The lesion appeared teal in colouration. Localised glandular ducts were irregular in shape and varied in size, with some showing fusion. Indentations within the lesions lacked visible glandular ducts. Microvascular structures were coarsened, twisted, and exhibited a grid-like distribution pattern (Fig. 2C, D).

**Figure 3.**
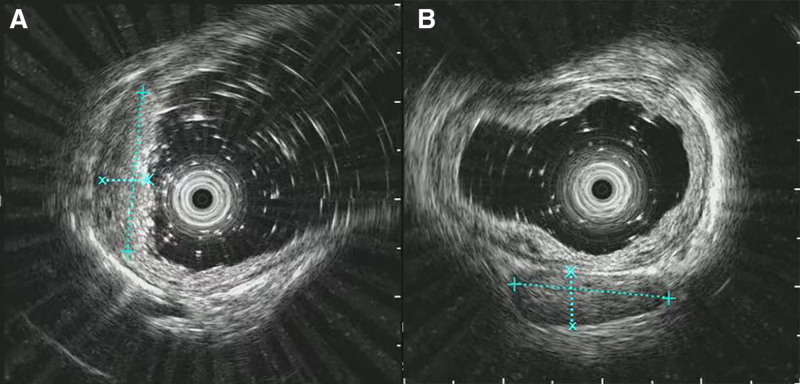
Endoscopic ultrasound findings. (A) Marked thickening of the mucosal layer; (B) enlarged lymph node detected in the periesophageal mediastinum.

Endoscopic ultrasonography (Fig. 3A, B): The lesion was located on the right posterior esophageal wall, approximately 29 to 31 cm from the incisors. The mucosal layer was notably thickened, with moderately low echogenicity and heterogeneous internal echogenicity. The submucosal layer remained intact at the lesion site. A perioesophageal mediastinal lymph node, measuring approximately 1.5 × 0.6 cm, was identified.

Enhanced computed tomography: Mild thickening of the lower esophageal wall and no enlarged lymph nodes were identified. The lymph nodes identified during endoscopic ultrasonography were consistent with reactive lymphadenopathy.

### 2.9. Preliminary diagnosis

1. Long-segment BE.
2. Flat-type esophageal lesion (0-IIb + 0-IIc) with high-grade adenomatous dysplasia and focal well-differentiated adenocarcinoma, suspected to invade the muscularis mucosae layer.

### 2.10. Treatment

**Figure 4.**
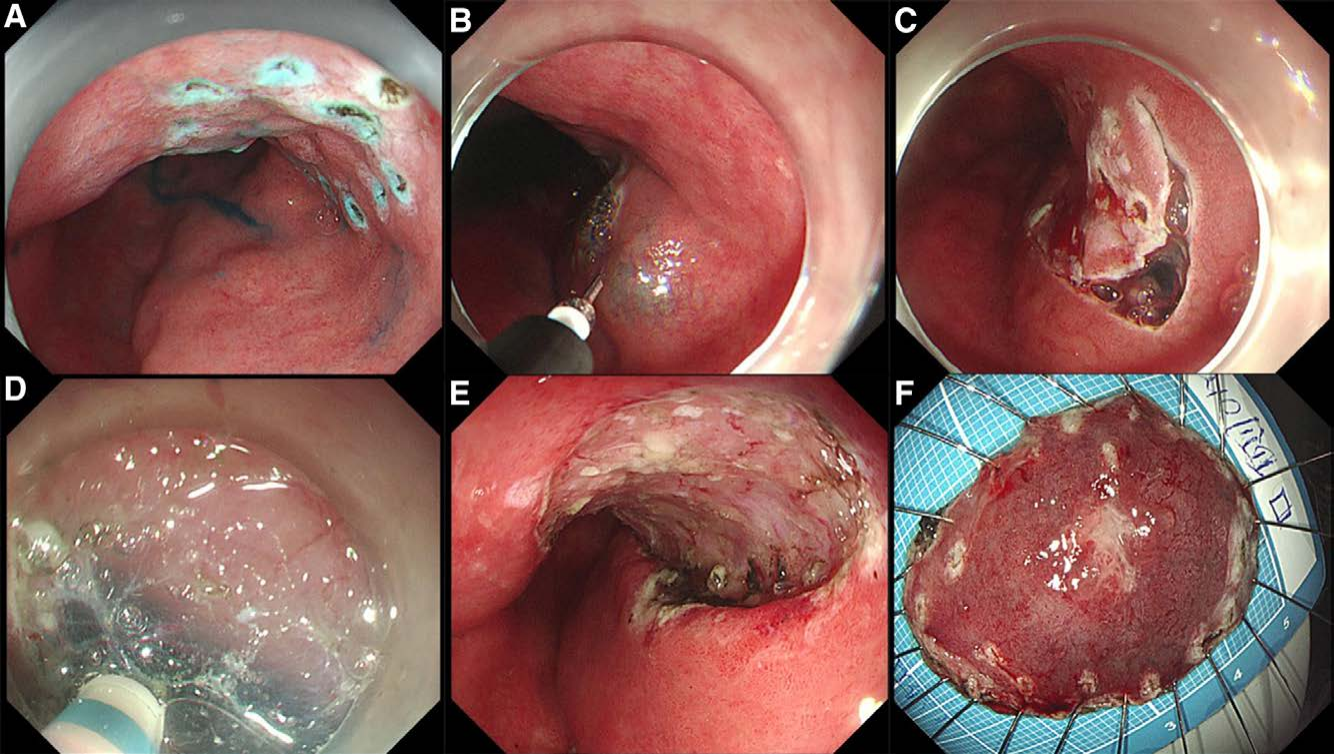
Surgical procedure. (A) Lesion marking; (B) submucosal injection; (C) circumferential pre-cutting; (D) submucosal dissection; (E) postoperative wound surface; (F) postoperative pathology specimen.

The patient had not received any treatment prior to surgery. Based on the patient’s medical history and relevant examinations, and in accordance with current guidelines and consensus, the patient was diagnosed with early-stage superficial esophageal cancer meeting the indications for ESD. Following preoperative evaluation, the ESD procedure was successfully performed under general anesthesia (Fig. 4A–F).

### 2.11. Outcome and follow-up

**Figure 5.**
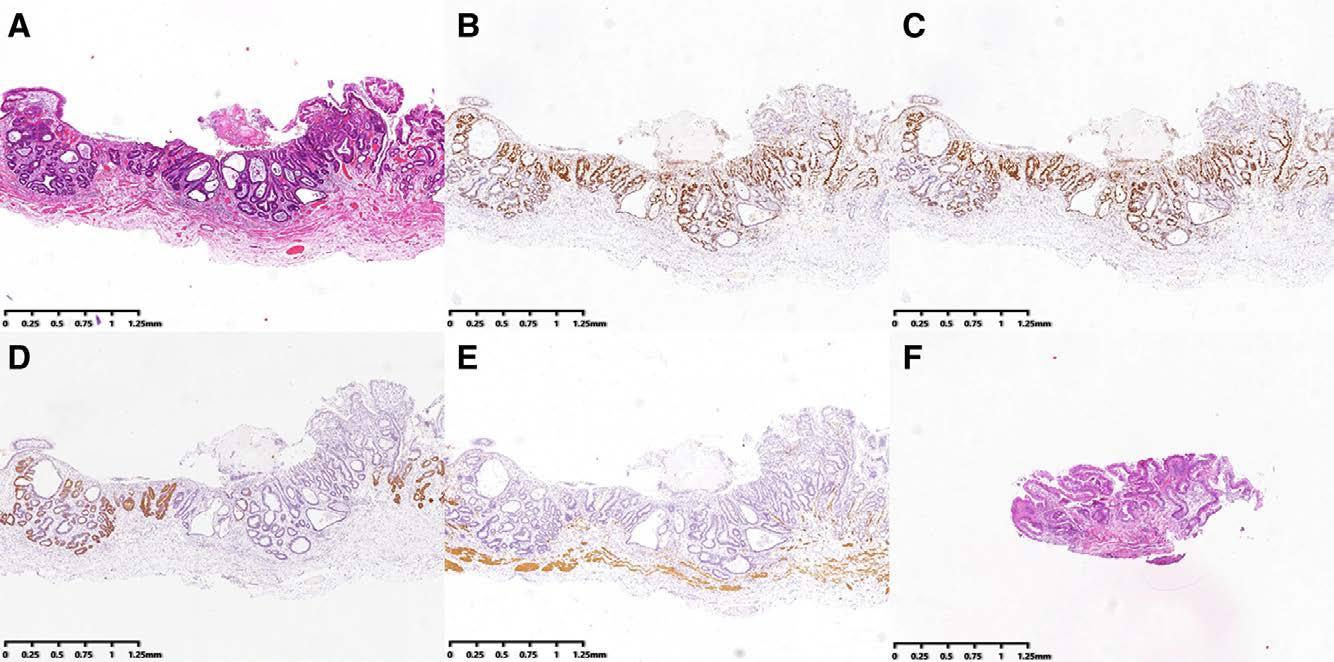
Postoperative pathological findings. (A) Marked cellular atypia with disordered glandular architecture and cancer cells infiltration into the muscularis mucosae, without penetration beyond it (hematoxylin-eosin stain, ×2); (B) Ki-67 (proliferation index + 30%; immunohistochemical stain, ×2); (C) Mucin-5AC(−) (immunohistochemical stain, ×2); (D) Mucin-6(−) (immunohistochemical stain, ×2); (E) Desmin (positive in smooth muscle), confirming cancer cell infiltration into the muscularis mucosae (immunohistochemical stain, ×2); (F) esophageal tissue exhibiting gastric mucosal metaplasia with, chronic atrophic mucositis and intestinalisation (hematoxylin-eosin stain, ×2).

Postoperatively, the patient exhibited no significant complications such as bleeding, pain, or vomiting. Supportive care was administered, including oxygen therapy, fluid resuscitation, anti-inflammatory therapy, mucolytic agents, acid suppression therapy, and nutritional support. The patient recovered well and was discharged without complications. Postoperative pathological examination revealed the following findings (Fig. 5A): Histological type: Early mucosal adenocarcinoma (moderately differentiated), 0-IIb + 0-IIc type. Specimen size: 1.2 × 0.6 × 0.4 cm. Invasion depth: Tumour infiltration reached the muscularis mucosae but did not penetrate it. Margins: Horizontal(−) and vertical(−), with the closest distance to the basal margin measuring 550 µm; no vascular invasion or perineural involvement was observed. Immunohistochemical findings (Fig. 5B–E): mucin MUC-5AC(−), MUC-6(−), tumor protein P53 (missense mutation), Ki-67 (proliferation index + 30%), caudal-type homeobox protein 2(+), carcinoembryonic antigen(−), desmin (positive in smooth muscle), smooth muscle actin (positive in smooth muscle), cluster of differentiation CD10(−), S-100(−), CD34(−), D2-40(−).

**Figure 6.**
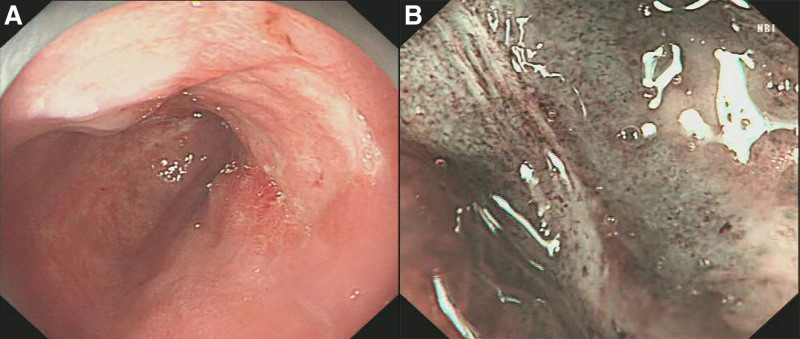
Three-month postoperative follow-up endoscopy. (A) Postoperative wound surface; (B) narrow-band imaging with magnifying endoscopy.

At the 3-month follow-up gastroscopy (Fig. 6A, B), an endoscopic examination revealed a post-ESD ulcer located on the right posterior esophageal wall, approximately 29 to 31 cm from the incisors. The ulcerated area appeared as a depressed erosion with a thin surface coating. NBI-ME showed a brownish discoloration at the lesion site, with the localized absence of glandular structures and dense, irregular microvascular patterns. Histopathological analysis (Fig. 5F) demonstrated chronic atrophic mucositis with intestinal metaplasia. Cytological examination performed 1 day prior to surveillance endoscopy revealed squamous hyperplasia on cytological analysis (Fig. 1B).

Six months after surgery, multiple small ulcers were observed near the ESD scar. Pathological biopsy revealed BE with chronic inflammation.

**Figure 7.**
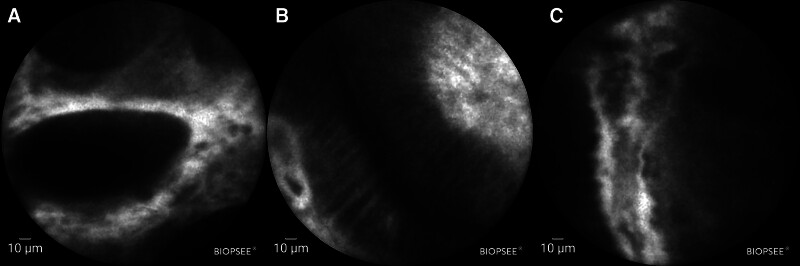
Confocal laser endomicroscopy. (A) Gastric epithelial cells are observed in the esophagus; (B) intestinal epithelial cells are observed in the esophagus; (C) tortuous and dilated microvascular structures in the esophagus.

CLE was performed on the patient during the third follow-up visit. Gastric epithelial cells and gastric pits were observed at the post-ESD scar site (Fig. 7A), accompanied by sodium fluorescein extravasation. Intestinal epithelial cells were identified in the scarred area with glands exhibiting distinct brush borders, though no goblet cells were detected (Fig. 7B). Tortuous and dilated microvascular structures were visualized in some lesional regions under examination (Fig. 7C).

## 3. Discussion

Long-segment Barrett’s esophagus (LSBE) represents an uncommon clinical entity in Asian populations, with LSBE complicated by adenocarcinoma demonstrating exceptional rarity. Through literature review, we identified that the overwhelming majority of reported cases (ranging from 15 to 22 cm in length with concomitant adenocarcinoma) originated from Western countries.^[4]^ While sporadic Japanese cases have been documented in Asia, none exhibited columnar-lined epithelium exceeding 10 cm in longitudinal extension.^[5,6]^ Notably, no LSBE cases with adenocarcinoma development have been formally documented in mainland China to date, rendering a 15-cm LSBE lesion with malignant transformation an extraordinarily rare presentation in this demographic. Recent epidemiological shifts, characterized by rapid socioeconomic development and Westernization of dietary patterns, have precipitated a marked increase in obesity prevalence and GERD incidence within China. This pathophysiological cascade consequently elevates the risk profile for BE progression and EAC development. These emerging trends necessitate heightened clinical vigilance and systematic documentation of therapeutic outcomes to facilitate evidence-based management protocols. The accumulation and dissemination of robust clinical data remain imperative for optimizing diagnostic algorithms and therapeutic interventions in this evolving disease paradigm.

In EAC derived from LSBE, the histological distribution of dysplasia and EAC tends to be multiple and diffuse.^[6]^ ESD is commonly used in China to treat superficial EAC in the Barrett’s esophagus. A study by Liu et al^[7]^ demonstrated that, for patients with T1b superficial esophageal cancer, ESD was associated with longer overall survival and progression-free survival compared to radical surgery. Similarly, a single-center study by Zhang et al^[8]^ found that, for patients with pT1b squamous esophageal cancer patients, ESD led to shorter hospital stays and lower complication rates than surgery. The horizontal tumor extent of the LSBE-related superficial EAC was accurately diagnosed using NBI-ME in the present case. Endoscopic ultrasonography was employed to delineate the depth of neoplastic infiltration, following which curative ESD was subsequently performed. The postoperative course was uneventful, with no procedure-related complications or endoscopic evidence of disease recurrence during longitudinal surveillance. This clinical outcome substantiates ESD as a viable therapeutic modality for early-stage EAC.

Esophageal cancer has a 5-year survival rate of approximately 20%,^[9]^ with early detection and treatment critical for improving prognosis. While endoscopic screening is effective, large-scale implementation is impractical, necessitating a more accurate and accessible alternative. The cytosponge™ device is an effective device. Gao et al^[10]^ combined it with artificial intelligence diagnostics for ESCC, achieving 90.0% sensitivity and 93.4% specificity.Subsequent studies confirmed its efficacy for detecting ESCC and esophagogastric junction adenocarcinoma.^[11]^ This minimally invasive approach enables cost-effective population-level screening, particularly valuable in regions with limited endoscopic resources. Despite its potential to identify high-risk populations for targeted endoscopic screening, larger clinical trials are needed to validate its clinical applicability. For this case, we used this detection method before and after surgery, and the cytological results were the same as those of pathology, indicating that the cytosponge™ device is effective for the detection of high-risk groups, and also provides case accumulation to prove the diagnostic efficacy of this method.

During the third follow-up examination, probe-based confocal laser endomicroscopy (pCLE) was performed and demonstrated no evidence of tumor recurrence. In recent years, the rapid advancement of medical optical imaging technology has enabled confocal microscopy imaging to emerge as a novel diagnostic approach for evaluating microstructural alterations in gastrointestinal lesions,^[12]^ owing to its superior spatial resolution and real-time imaging capability. This technology shows potential for enhancing clinical decision-making through more precise personalized therapeutic strategies and establishing optimized follow-up protocols.

Although case reports are inherently limited by their small sample size, this particular case, due to the rare occurrence within the Chinese population and the distinctive nature of its management and diagnostic workup, offers valuable insights for clinical practice. It effectively underscores the potential clinical utility of the cytosponge™ device and confocal endomicroscopy, providing significant educational value for enhancing diagnostic recognition and therapeutic strategies.

## 4. Conclusion

ESD is an effective treatment for early-stage esophageal adenocarcinoma, early detection and early treatment is the key to prolong the survival of patients, cytosponge™ device helps to detect high-risk groups. CLE represents an advanced endoscopic imaging modality that facilitates preoperative diagnosis and postoperative surveillance in early-stage esophageal carcinoma.

## Author contributions

**Conceptualization:** HaoQiang Zhi, HuiFang Pang.

**Data curation:** HaoQiang Zhi, Miao Wang.

**Investigation:** Ling Sun, Wenjing Ao.

**Visualization:** HaoQiang Zhi, WeiJian Ju.

**Writing – original draft:** HaoQiang Zhi.

**Writing – review & editing:** Quan Man, HuiFang Pang.
